# Assessing the reliability of FTIR spectroscopy measurements and validity of bioelectrical impedance analysis as a surrogate measure of body composition among children and adolescents aged 8–19 years attending schools in Kampala, Uganda

**DOI:** 10.1186/s12889-018-5627-y

**Published:** 2018-06-04

**Authors:** Catherine T. Ndagire, John H. Muyonga, Dan Isabirye, Benard Odur, Serge M. A. Somda, Richard Bukenya, Juan E. Andrade, Dorothy Nakimbugwe

**Affiliations:** 1 School of Food Technology, Nutrition and Bio-engineering, Makerere University, Kampala, Uganda; 2 Department of Biochemistry and Sports Science, Makerere University, Kampala, Uganda; 3 Department of Statistical Methods and Actuarial Science, School of Statistics, Makerere University, Kampala, Uganda; 4 Centre MURAZ, Bobo-Dioulasso, Burkina Faso; 5 Division of Nutritional Sciences, University of Illinois, Urbana-Champaign, USA; 6 Department of Food Science and Human Nutrition, University of Illinois, Urbana-Champaign, USA

**Keywords:** Body composition, Bioelectric impedance analysis, Deuterium dilution method, Children, Adolescents, agreement, reliability

## Abstract

**Background:**

Accurate measurement of body composition in children and adolescents is important as the quantities of fat and fat-free mass have implications for health risk. The objectives of the present study were: to determine the reliability of Fourier Transform Infrared spectroscopy (FTIR) measurements and; compare the Fat Mass (FM), Fat Free Mass (FFM) and body fat percentage (%BF) values determined by bioelectrical impedance analysis (BIA) to those determined by deuterium dilution method (DDM) to identify correlations and agreement between the two methods.

**Methods:**

A cross-sectional study was conducted among 203 children and adolescents aged 8–19 years attending schools in Kampala city, Uganda. Pearson product-moment correlation at 5% significance level was considered for assessing correlations. Bland Altman analysis was used to examine the agreement between of FTIR measurements and between estimates by DDM and BIA.. Reliability of measurements was determined by Cronbach’s alpha.

**Results:**

There was good agreement between the in vivo D_2_O saliva enrichment measurements at 3 and 4 h among the studied age groups based on Bland-Altman plots. Cronbach’s alpha revealed that measurements of D_2_O saliva enrichment had very good reliability. For children and young adolescents, DDM and BIA gave similar estimates of FFM, FM, and %BF. Among older adolescents, BIA significantly over-estimated FFM and significantly under-estimated FM and %BF compared to estimates by DDM. The correlation between FFM, FM and %BF estimates by DDM and BIA was high and significant among young and older adolescents and for FFM among children.

**Conclusions:**

Reliability of the FTIR spectroscopy measurements was very good among the studied population. BIA is suitable for assessing body composition among children (8–9 years) and young adolescents (10–14 years) but not among older adolescents (15–19 years) in Uganda. The body composition measurements of older adolescents determined by DDM can be predicted using those provided by BIA using population-specific regression equations.

## Background

Nutrition-related non-communicable diseases (NCDs) such as hypertension, high blood glucose and cholesterol levels, diabetes and cardiovascular diseases, are increasing and are predicted to be the major cause of morbidity and mortality in most developing nations by 2020 [1]. In children and adolescents, the most common risk factors for nutrition-related NCDs include overweight, obesity, physical inactivity and unhealthy diets [2]. Pediatric and adolescent overweight and obesity are the driving force behind metabolic syndrome risk that has become a growing public health concern in low and middle-income countries (LMICs) [3]. This calls for interventions to prevent and manage childhood and adolescent overweight and obesity. These interventions’ design, monitoring and evaluation rely on correct identification of overweight and obese individuals. Therefore, there is need for accurate body composition measures to correctly identify overweight and obese individuals.

Body mass index (BMI) is the commonly used technique to determine nutrition status because it is inexpensive, fast and non-invasive [4]. However, it is a poor index of fatness and has poor sensitivity and inaccuracy for categorizing of obesity and overweight [5]. These limitations make BMI a poor outcome for research on the efficacy of nutrition programs. A number of reference methods are used to estimate body composition, including underwater weighing (UWW) technique, air displacement plethysmography (ADP), dual-energy X-ray absorptiometry (DEXA) and Deuterium Dilution Method (DDM) [6]. While DDM has widely been used due to its simplicity and relatively low cost, no published study was found on FTIR spectroscopy measurements’ reliability among any population in Uganda. Reliability is defined as the degree of consistency and the lack of error in a measurement [7]. Despite the scarcity of studies on the reliability of FTIR measurements, it is a prerequisite for investigators aiming to validate a device or technique to evaluate the reliability of the reference method, as lack of reliability often masks the actual effects and leads to misinterpretation [8]. Internal reliability which measures repeatability of a tool is determined by Cronbach’s alpha [7] and by Bland-Altman analysis [9].

BIA is a rapid, cheap, safe and simple technique for measuring body composition both in the field and in clinical settings [10], based on population-specific predictive equations [11]. Since the validity of BIA measurements varies with age and ethnicity [12], a number of studies have assessed the validity of BIA devices in various populations of children and adolescents commonly using DEXA and DDM as reference techniques [13–16]. However, BIA’s validity for assessment of body composition and agreement with reference techniques like DDM has not been assessed among any population in Uganda, including children and adolescents. Therefore, the objectives of the present study were to: i) assess the reliability of FTIR spectroscopy measurements of saliva D_2_O enrichment for determination of body composition and; ii) compare the body composition variables determined by BIA and DDM and, identify possible correlations and agreement between the two methods.

## Methods

### Subjects

In a cross-sectional study, 203 apparently healthy (based on self-proclamation) participants attending primary and secondary schools in Kampala city, Uganda were selected through a two-stage cluster sample design. The Ministry of Education and Sports provided an up to date list of all the primary and secondary schools in Kampala from which schools to participate in the study were randomly selected. Due to homogeneity between schools and between students in divisions of Kampala, schools were treated as clusters. Sampling of students from schools followed probability proportion to size procedure and a sample of 203 participants aged 8–19 years was randomly selected using random numbers.

Since sample size determination for validation studies is rarely ever justified a priori [17], for this study validation sample size was based on recommendations of researchers in the field of validity studies and from sample sizes used in previous validity studies as stated below. For a study of agreement between two methods of measurement, a sample size of 100 subjects is sufficient, giving a 95% CI of about +/− 0.34 *s,* where *s* is the standard deviation of the differences between measurements by the two methods*.* A sample of 200 subjects is better since it gives a 95% CI of about +/− 0.24 *s* [18]. A sample size of 100 to 200 subjects is a reasonable size for validation studies as it’s adequate for a range of likely degrees of validity and allows for appropriate deletion of some subjects [19]. Furthermore, a minimum of 80 subjects for validity studies provides highly representative estimates of the main study samples [20]. For most studies, sample sizes used have often been small, ranging from 15 to 189 subjects [21–25]. Against this background, for this study, 203 participants were selected. At least four subjects were targeted for each age. The subject to item ratio (*n* = 4) is the frequently recommended approach when performing an exploratory factor analysis [17]. In a similar study to assess body composition in Mexican school children of different geographical regions and ethnicity, two children per age and ethnic group were regarded as sufficient [26].

The selected subjects’ nutritional status was evaluated by anthropometric measurements: BMI, waist circumference, waist to hip ratio and weight to height ratio and their body composition was assessed by BIA and DDM. Immediately after the anthropometric and BIA measurements were taken, saliva samples were collected from the subjects and D_2_O doses were given to them. This permitted the assessments to be performed at the same time and under the same conditions, with a consequent constant state of hydration during all methods of body composition assessment used in the study.

### Assessing height and weight

Height and weight were taken by trained researchers using standard equipment. Body weight was measured to the nearest 0.1 kg using a weighing scale, (Seca 899; Seca Weighing and Measuring Systems, Model No. 8691321004, SECA Gmbh & Co. Germany made in China) with minimal clothing and no shoes. Height was measured to the nearest 0.1 cm using a height board (Shorr-board, height board, Weight and Measure LLC, Irwin J. Shorr, MPH, MPS. Olney, Maryland USA) without shoes. BMI (kg/m^2^) was calculated as weight in kilogram divided by the square of height in meters.

### Assessing waist and hip circumferences

Waist circumference (WC) was measured to the nearest 0.1 cm in standing position at the midpoint between the lowest rib and the iliac crest and at the end of normal expiration, using a measuring tape. Hip circumference (HC) was measured to the nearest 0.1 cm in standing position at the widest point of the hips using a measuring tape (Lufkin Executive Diameter Steel Tape, 2 m Thinline Model W606 PM, Apex Tool Group, LLC NC 27502, USA).

### Body composition assessment by bioelectrical impedance analysis

Body composition by BIA was measured using a BIA (Tanita SC-331S Body Composition Analyzer; Tanita Inc., Arlington Heights, IL) instrument, which provides a measure of fat mass and fat-free mass using in-built manufacturers’ equations. Impedance was measured with the subject standing barefoot on the metal foot-plates of the machine for approximately 1 min. The subject’s age, gender, and height were entered into the machine, and a standard 0.5 kg was entered as an adjustment for clothing weight for all participants.

### Body composition assessment using deuterium dilution technique

A baseline saliva sample was collected from participants 2 hours after their last meal. Each participant then received an oral dose of D_2_O (0.5 g/kg body weight). Two endpoint saliva samples were collected at 3 and 4 h after D_2_O dose ingestion. Samples were stored in plastic saliva vials at − 20 °C until they were analyzed for D_2_O using FTIR spectroscopy instrument (FTIR-8400S, Shimadzu Corporation, Japan) according to manufacturer’s instructions. The instrument was housed in the Department of Biochemistry, Makerere University Kampala, Uganda. The instrument settings were: measurement mode: absorbance; apodization: square triangle; number of scans: 32; resolution: 2.0 and; range (cm^− 1^): minimum 2300 - maximum 2900.

A ‘background’ scan was performed using the unenriched drinking water that was used to make the calibration standard (zero standard) and the instrument was calibrated using a prepared D_2_O standard (1000 mg/kg). Total body water (TBW) was calculated from the saliva sample by plateau method, based on the assumption that this plateau was reached at 3 or 4 h. FM and %BF were estimated from TBW while FFM was calculated from FM.

### Statistical analysis

Descriptive statistics (means and confidence intervals) were used for presentation of measurements data for D_2_O enrichment, participants’ characteristics and body composition (FFM, FM, and %BF) by DDM and BIA. Normality of variables was inspected visually using normal histogram plots. Box plots were used to inspect for data outliers 8 of which were removed. To show the relationship between saliva D_2_O enrichment at 3 and 4 h after ingestion of the D_2_O dose when equilibration is achieved, Pearson product-moment correlation was used. Reliability of the two FTIR measurements was verified using the Bland-Altman analysis by plotting the differences between the two measurements of each subject against the mean value of the two measurements. Mean differences and limits of agreement were determined according to Bland Altman procedures. Limits of agreement were considered as the mean of differences between the measurements at 3 and 4 h ± 1.96 × their standard deviation. Cronbach’s alpha was used to assess the level of reliability of the FTIR spectroscopy measurements at 3 and 4 h after D_2_O dose ingestion. Cronbach’s α values between 0.7–0.9 were considered representative of good reliability, while values above 0.9 were considered representative of very good reliability [27].

Paired t-tests were used to compare mean measures of FFM, FM, and %BF by BIA and DDM. To show the relationship between DDM and BIA, Pearson product-moment correlation was considered. The Bland Altman plots examined the agreement between DDM and BIA for measuring FFM, FM, and %BF. Mean differences and limits of agreement were calculated according to Bland Altman procedures. Limits of agreement were considered as the mean of differences between measurements by DDM and BIA ± 1.96 × their standard deviation. The analyses were done using with STATA version 13 software and the level of significance was set at *P* < 0.05.

## Results

There were wide ranges for body weight, height, BMI, waist circumference and hip circumference across the different age groups (Table 1) In the current study, 16 children aged 8–9 years, 112 young adolescents aged 10–14 years and 67 older adolescents aged 15–19 years; 84 males and 111 females with mean (95% confidence interval) age 13.44 (12.98 to 13.90) years, weight 44.61 (42.92 to 46.31) kg, height 1.51 (CI: 1.50, 1.53) m, waist circumference 65.87 (CI: 65.02, 66.71) cm and hip circumference 82.48 (CI: 80.97, 83.99) cm participated.

**Table 1:** Participants’ characteristics

		Mean (95% Confidence Interval)		
Characteristic	Children (8–9 years)	Young adolescents (10–14 years)	Older adolescents (15–19 years)	Overall
N	16	112	67	195
Male	7	51	26	84
Female	9	61	41	111
Age	8.34 (8.11 to 8.64)	11.80 (11.57 to 12.04)	17.39 (17.09 to 17.69)	13.44 (12.98 to 13.90)
Weight (kg)	28.31 (25.88 to 30.75)	40.40 (38.74 to 42.05)	55.55 (53.48 to 57.62)	44.61 (42.92 to 46.31)
Height (m)	1.31 (1.28 to 1.35)	1.48 (1.47 to 1.50)	1.62 (1.60 to 1.64)	1.51 (1.50 to 1.53)
BMI (kg/m^2^)	16.30 (15.65 to 16.95)	18.16 (17.69 to 18.63)	21.23 (20.53 to 21.93)	19.07 (18.64 to 19.50)
Waist circumference (cm)	58.56 (56.83 to 60.28)	65.07 (64.06 to 66.08)	68.94 (67.62 to 70.26)	65.87 (65.02 to 66.71)
Hip circumference (cm)	68.43 (66.25 to 70 .60)	79.20 (77.70 to 80.71)	91.32 (89.17 to 93.47)	82.48 (80.97 to 84.26)
Waist height ratio	0.44 (0.44 to 0.46)	0.44 (0.43 to 0.44)	0.43 (0.42 to 0.44)	0.44 (0.43 to 0.44)
Waist hip ratio	0.86 (0.84 to 0.88)	0.82 (0.82 to 0.83)	0.76 (0.74 to 0.78)	0.80 (0.80 to 0.81)
3 h deuterium enrichment (ppm)	722.86 (626.83 to 818.88)	796.77 (772.52 to 821.03)	790.85 (744.72 to 836.99)	788.67 (766.49 to 810.86)
4 h deuterium enrichment (ppm)	720.06 (622.18 to 817.94)	799.78 (775.74 to 823.83)	798.69 (753.26 to 844.11)	792.87 (770.84 to 814.89)
DDM Total body water (litres)	17.18 (15.90 to 18.47)	24.97 (24.07 to 25. 87)	32.05 (30.75 to 33.35)	26.76 (25.84 to 27.68)
DDM Total body water (%)	60.92 (59.38 to 62.47)	62.29 (61.44 to 63.14)	58.25 (56.21 to 60.28)	60.79 (59.90 to 61.68)
DDM Fat free mass (kg)	23.48 (21.72 to 25.23)	34.11 (32.88 to 35.34)	43.78 (42.00 to 45.56)	36.56 (35.30 to 37.82)
BIA Fat free mass (kg)	24.13 (22.10 to 26.16)	33.89 (32.72 to 35.06)	46.62 (45.05 to 48.19)	37.46 (36.13 to 38.79)
DDM Fat mass (kg)	4.84 (3.90 to 5.77)	6.29 (5.61 to 6.96)	11.77 (9.97 to 13.58)	8.05 (7.23 to 8.87)
BIA Fat mass (kg)	4.18 (3.48 to 4.88)	6.51 (5.80 to 7.22)	8.93 (7.54 to 10.32)	7.15 (6.50 to 7.81)
DDM Fat (%)	16.77 (15.65 to 16.95)	14. 90 (13.74 to 16.06)	20.43 (17.64 to 23.71)	16.95 (15.74 to 18.17)
BIA Fat (%)	14.61 (12.83 to 16.39)	15.34 (14.21 to 16.45)	15.42 (13.32 to 17.52)	15.30 (14.34 to 16.27)
Impedance	660.00 (626.16 to 693.84)	596.63 (581.35 to 611.90)	515.04 (500.91 to 529.16)	573.79 (561.67 to 585.92)

Cronbach’s alpha values for the two measurements of saliva D_2_O enrichment were high (0.999, 0.997 and 0.996 for children, young and older adolescents, respectively) (Table 2).

**Table 2:** Cronbach’s alpha values of the two readings for the different age groups

Age category	Cronbach’s Alpha
8–9 years	0.999
10–14 years	0.997
15–19 years	0.996

**Fig. 1:**
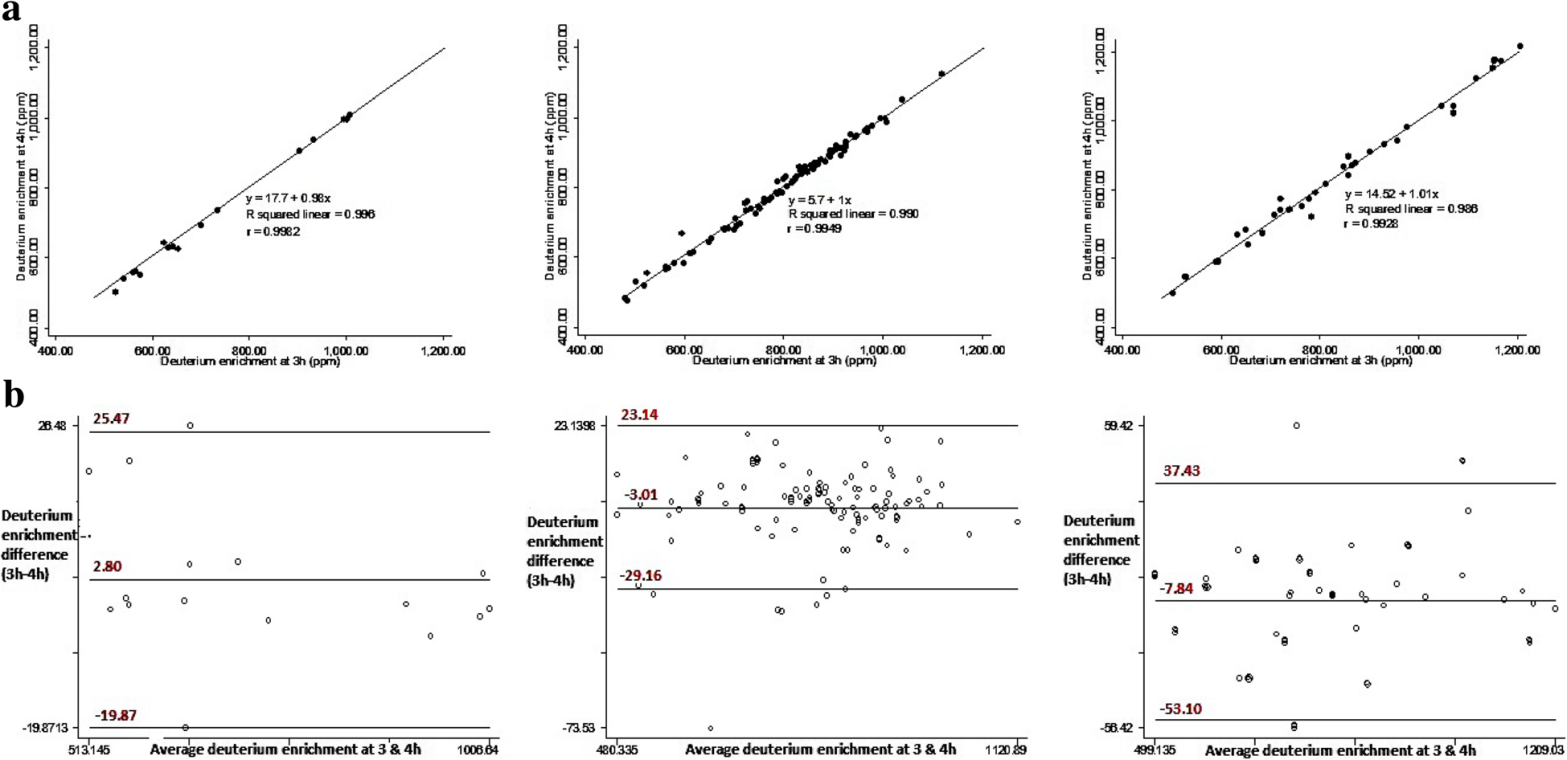
Regression and Bland-Altman plots for saliva D_2_O enrichment among children (left), young adolescents (middle) and older adolescents (right)

**Fig. 2:**
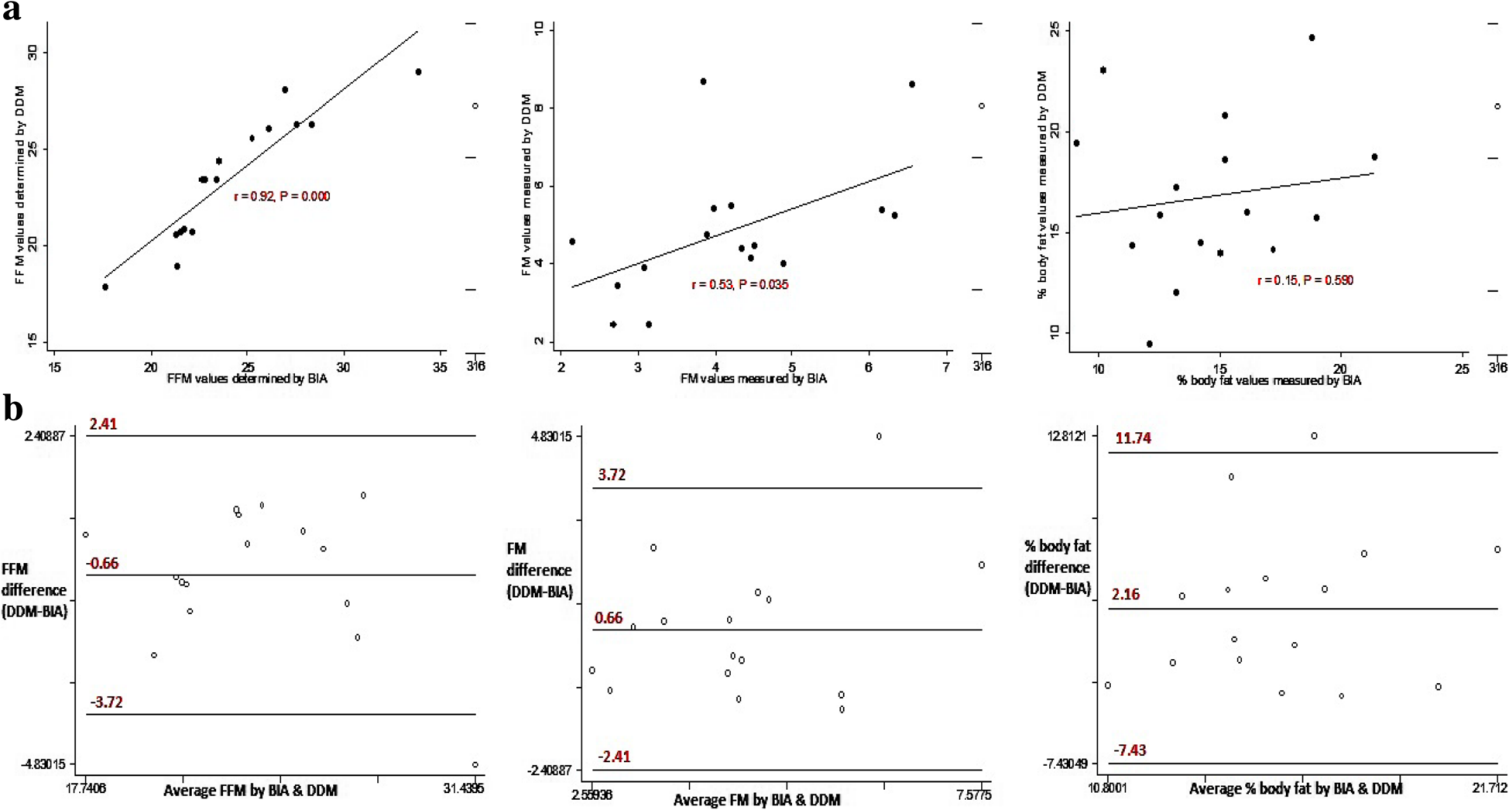
Regression and Bland-Altman plots for FFM (left), FM (middle) and % body fat (right) determined by DDM and BIA among children

**Fig. 3:**
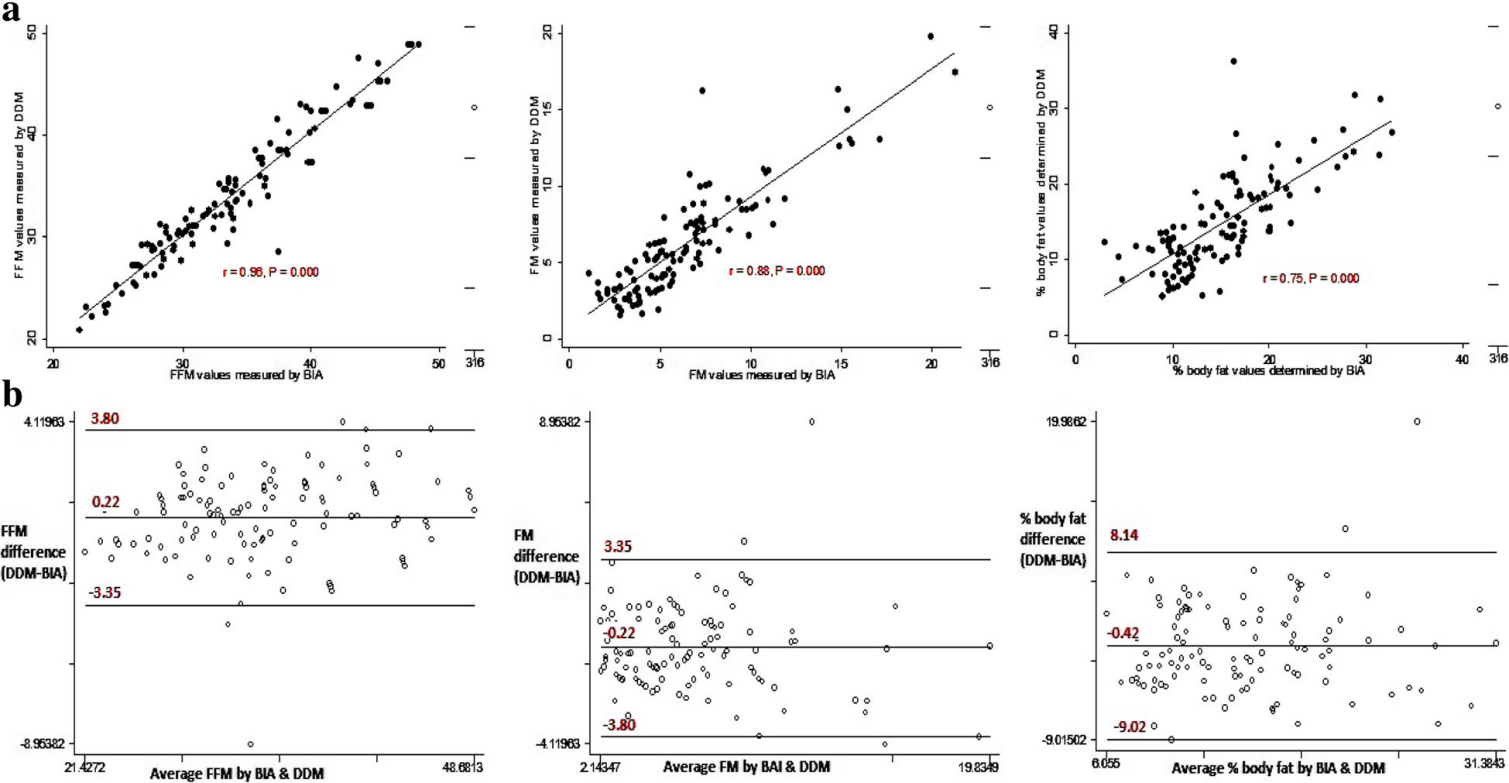
Regression and Bland-Altman plots for FFM (left), FM (middle) and % body fat (right) determined by DDM and BIA among young adolescents

**Fig. 4:**
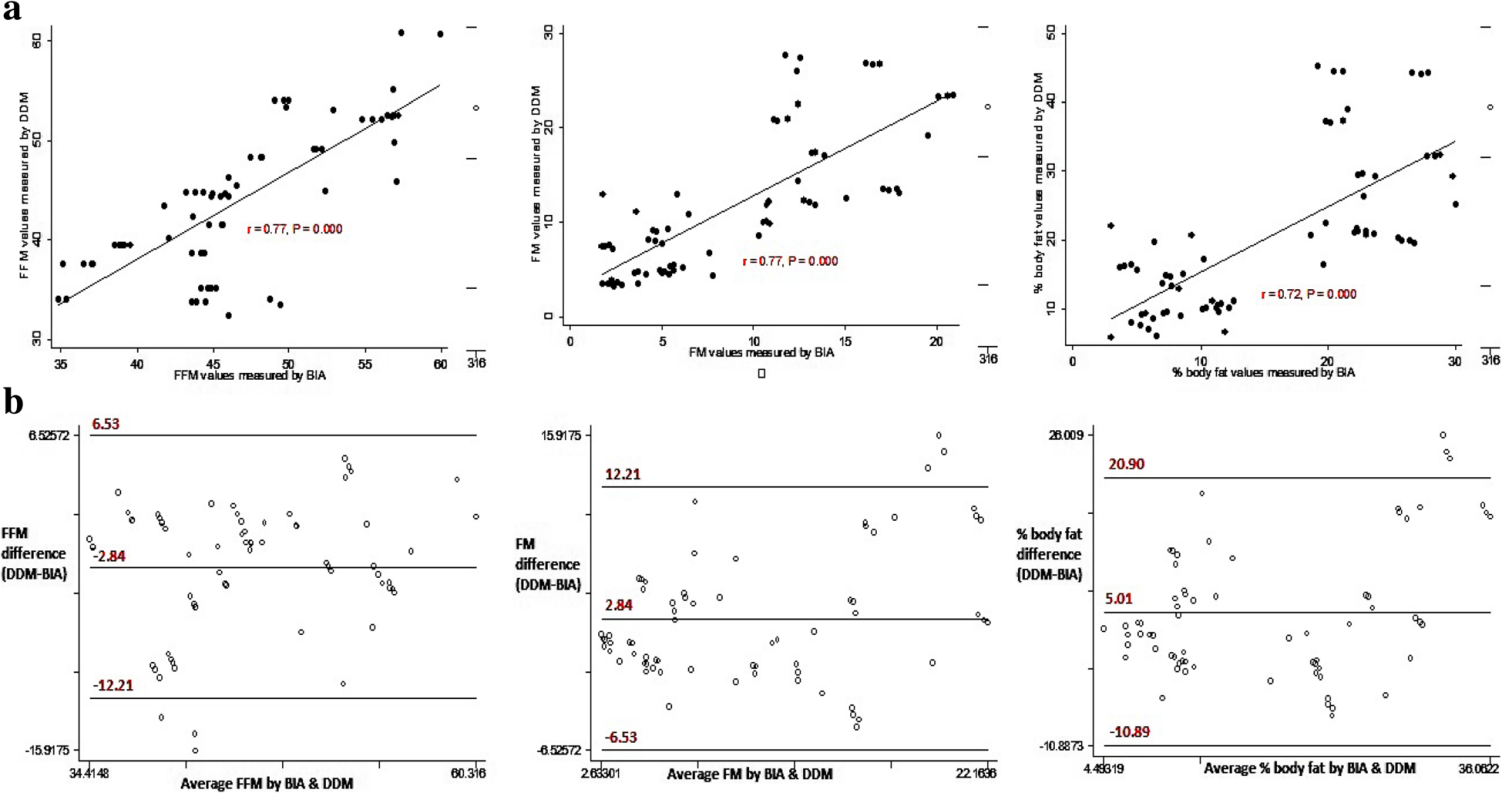
Regression and Bland-Altman plots for FFM (left), FM (middle) and % body fat (right) determined by DDM and BIA among old adolescents

The correlation coefficients for deuterium enrichment at 3 and 4 h were high and positive among children, young and older adolescents at *r* = 0.998, 0.995, and 0.993 respectively (Fig. 1a). The Bland-Altman plots showed random nature of spread with no detectable proportional bias for saliva D_2_O enrichment at 3 and 4 h among the different age groups (Fig. 1b). For children and young adolescents, FFM, FM and %BF estimates by DDM were not statistically significantly different from those measured by BIA (Table 3). Among older adolescents, DDM significantly underestimated FFM (*P* < 0.0001) and significantly overestimated FM and %BF at P < 0.0001 and P < 0.0001 respectively compared to BIA. Among young and older adolescents, the correlations between FFM, FM and % BF estimates by DDM and BIA were high and significant at *r* > 0.7 and P < 0.0001 (Figs. 3a and 4a). The Bland-Altman plots for FFM, FM, and %BF showed a random nature of spread with no detectable significant negative bias for FFM, FM and % BF values estimated by DDM and BIA among the different age groups (Figs. 2b, 3b and 4b).

**Table 3:** Body composition mean values (CI), mean difference (CI) and *P*-values between DDM and BIA among different age groups

	Body composition means			
	DDM	BIA	Mean difference	*P*-value
Children (8–9 years)				
FFM (kg)	23.48 (21.72–25.23)	24.13 (22.10–26.16)	−0.657 (−1.474–0.160)	0.1071
FM (kg)	4.84 (3.90–5.77)	4.18 (3.48–4.88)	0.657 (− 0.160–1.474)	0.1071
%BF	16.77 (14.66–18.88)	14.61 (12.83–16.39)	2.157 (−0.397–4.711)	0.0920
Young adolescents (10–14 years)				
FFM (kg)	34.11 (32.88–35.34)	33.89 (32.72–35.06)	0.224 (− 0.111–0.559)	0.1876
FM (kg)	6.29 (5.61–6.96)	6.51 (5.80–7.22)	−0.224 (− 0.559–0.111)	0.1876
%BF	14. 90 (13.74–16.06)	15.34 (14.21–16.45)	−0.436 (− 1.239–0.367)	0.2846
Older adolescents (15–19 years)				
FFM (kg)	43.78 (42.00–45.56)	46.62 (45.05–48.19)	−2.841 (− 3.983 - -1.699)	< 0.0001
FM (kg)	11.77 (9.97–13.58)	8.93 (7.54–10.32)	2.841 (1.699–3.983)	< 0.0001
%BF	20.43 (17.64–23.71)	15.42 (13.32–17.52)	5.006 (3.068–6.944)	< 0.0001

DDM and BIA exhibited generally narrower limits of agreement for FFM, FM or % BF among children and young adolescents than among older adolescents (Fig. 2b, 3b, and 4b). Older adolescents (15–19-years) exhibited the largest mean differences for FFM (− 2.84 kg), FM (2.84 kg), and %BF (5.01) while young adolescents (10–14-years) exhibited lowest mean differences for FFM (0.22 kg), FM (− 0.22 kg) and %BF (− 0.44) (Table 3). Furthermore, the mean differences between DDM and BIA for measures of FFM, FM and %BF for older adolescents exhibited largest 95% confidence intervals compared to those for children and young adolescents. The Bland Altman plots for FFM, FM, and %BF for older adolescents exhibited largest limits of agreement compared those of children and young adolescents (Fig. 2b, 3b and 4b).

## Discussion

Prior to this work, no published studies were found on the reliability of FTIR saliva D_2_O enrichment measurements among populations in Uganda. In this study, the reliability of FTIR spectroscopy measurements among children and adolescents in Uganda was assessed. The high and positive correlation between 3 and 4-h FTIR spectroscopy measurements is indicative of similarity and reproducibility of the two sets of measurements. The Bland-Altman plots that showed no apparent trend in error differences between the measurements taken after 3 and those taken after 4 h imply that saliva D_2_O enrichment measurements were reproducible among the study population. The high Cronbach’s alpha value (> 0.9) among all studied age groups indicates very good repeatability of the FTIR spectroscopy saliva D_2_O enrichment measurements among children, young and older adolescents in Uganda. The FTIR spectroscopy instrument can, therefore, provide reliable measures for D_2_O saliva enrichment and thus suitable for validation of other body composition assessment techniques for more accurate assessment of body composition among children and adolescents in Uganda. Furthermore, the FTIR spectroscopy technique has several advantages in assessing body composition including simplicity to carry out, minimal subject cooperation requirements, acceptability in all age groups [28], non-invasiveness, relatively low cost, easy administration of tracers, and easy collection of samples [29].

In this study, the ability of the inbuilt equations from the Tanita SC-331S BIA instrument to assess body composition of children and adolescents in Uganda by using DDM as a reference method also was investigated. Prior to this work, no published studies were found comparing the body composition estimates obtained by BIA to those obtained by DDM among children and adolescents in Uganda. Since estimates for body composition had varying agreement across the studied age groups, DDM and BIA are generally not interchangeable across children and adolescents in Uganda. The none-statistically significantly different (*P* > 0.05) FFM, FM and %BF measures by DDM and BIA among children and young adolescents imply possibility for agreement between the two methods in these age categories. For children and young adolescents, the generally narrow limits of agreement, the small mean discrepancies (biases) for the FFM, FM and %BF estimates and their narrow 95% confidence intervals of means imply that DDM and BIA estimates for FFM, FM, and %BF agree and can be used interchangeably for either FM, FFM, or %BF for these age categories in Uganda. These findings are similar to those by Mehta and others who found agreement between BIA and DDM for FFM, FM and %BF among children 14 years of age or younger with Intestinal Failure [23]. In a study to validate 2 portable BIA devices; the Inbody 230 and the Tanita BC-418 for body composition assessment in healthy Taiwanese school-age children, Bland-Altman analysis showed clinically acceptable agreement between the Inbody 230 device and DEXA for FFM measurements [15].

On the other hand, the statistically significantly different mean values (*P* < 0.05) for FFM, FM and %BF among older adolescents imply no possibility for agreement between the two methods. The wide limits of agreement for FFM, FM, or %BF exhibited by Bland Altman plots for older adolescents, the big mean discrepancies (biases) for the FFM, FM and %BF estimates and their wide 95% confidence intervals in this age group imply limited agreement between the two methods. This reveals that DDM and BIA are not directly interchangeable for either FM, FFM, or %BF among older adolescents (15–19 years) in Uganda. Similar to this study’s findings where BIA overestimated FFM among older adolescent and underestimated their FM and %BF are those by Resende and others who reported that BIA overestimated the measures of FFM and underestimated the measures of FM compared to those provided by DDM among obese adolescents in Brazil [5]. Resende and others reported a high, positive and significant correlation between FFM and FM values determined by DDM and BIA but there was no agreement between the two methods among obese adolescents [5] as was the case for older adolescents in this study. In a study to validate predictive equations of BIA to FFM estimation in army cadets aged 17–24 years, Langer and others observed significant differences between FFM values from 8 predictive BIA equations and no good agreement with DXA [11] Also, among healthy Indian children and adolescents aged 5–18 years, there was no agreement between BIA and DXA in assessment of body composition [13]. A possible explanation for the discrepancy of body composition among older adolescents by the BIA system’s inbuilt prediction equations is that they are normally based on Western European or North American populations, which may differ in body composition and proportion when compared to the population under study [30]. Growth involves the deposition of both fat mass (FM) and fat-free mass (FFM) components and human body composition is ethnicity dependent [31]. No literature was found regarding the age- and sex-related pattern of changes in body composition for populations in Uganda.

While the study was the first of its kind among populations in Uganda, it was not without limitations. DDM was used as the reference method which, although widely validated as a reliable estimate, is not a gold standard for body composition. Ideally, a four-component model would have been used as the reference method, but this was not possible in our study setting. While DDM has the advantage that it is relatively easy to perform, it is not without limitations: one assumption is the hydration of FFM, which may vary among persons by age, sex, maturation and ethnicity and to estimate FFM from TBW, age, and sex-specific hydration fractions were used [6]. But the hydration of FFM values used for computation of TBW to estimate FFM, FM, and %BF were not Uganda specific. Higher hydration factors have been observed among African American adults compared to whites using a four-component model [32]. However, there is no information on the hydration factors of FFM for Ugandan populations.

## Conclusions

The reliability of the FTIR spectroscopy saliva D_2_O enrichment measurements was very good among the studied population. This technique can be used as a reference technique in the validation of field techniques like BIA for more accurate estimation of body composition in resource-poor countries that cannot afford four-compartment (gold standard) techniques.

The other results of the study showed that DDM and BIA can be used interchangeably for FFM, FM, and %BF for children and young adolescents aged 8–14 years in Uganda but not interchangeable for the assessment of body composition in older adolescents aged 15–19 years in Uganda. For that reason, among older adolescents in Uganda, BIA is not a valid measure for body composition, so deriving population-specific BIA equations may be a suitable approach for assessing body composition. The study, therefore, revealed BIA’s limitations in assessing body composition among children and adolescents in Uganda.

## Abbreviations

%BF: Body fat percentage
^2^H: Deuterium
ADP: Air displacement plethysmography
BIA: Bioelectrical impedance analysis
BMI: Body mass index
D_2_O: Deuterium oxide
DDM: Deuterium dilution method
DEXA: Dual-energy x-ray absorptiometry
FFM: Fat-free mass
FM: Fat mass
FTIR: Fourier transform infrared
IAEA: International atomic energy agency
LMICs: Low and middleiIncome countries
NCDs: Non-communicable diseases
TBW: Total body water
UWW: Underwater weighing
VD: Dilution space
WHO: World Health Organization
